# Supplementary material for: The Use of Pb Isotope Ratios to Determine Environmental Sources of High Blood Pb Concentrations in Children: A Feasibility Study in Georgia
Source: Int J Environ Res Public Health. 2022 Nov 15;19(22):15007. doi: 10.3390/ijerph192215007 (PMC9690981; doi:10.3390/ijerph192215007)
Supplement: Supplementary file 1 [file ijerph-19-15007-s001.zip › ijerph-1999583-supplementary.pdf]

Pb isotope ratios to link environmental sources with high blood Pb concentrations in children: a feasibility study in Georgia

Supplementary Information

Adam Laycock[1*], Simon Chenery[2], Elizabeth Marchant[1], Helen Crabbe[1], Ayoub Saei[3], Ekaterine Ruadze[4], Michael Watts[2], Giovanni S. Leonardi[1,5], Tim Marczylo[1]

1 UK Health Security Agency, Chemical, Radiation and Environmental Hazards Directorate, Harwell Campus, Chilton, OX11 0RQ, UK

2 British Geological Survey, Kingsley Durham Centre, Keyworth, Nottingham, NG12 5GG, UK

3 UK Health Security Agency, Colindale, London, UK

4 The National Center for Disease Control and Public Health, #99 Kakheti Highway, Tbilisi, Georgia

5 Department of Public Health, Environments and Society, London School of Hygiene and Tropical Medicine, London, WC1E 7HT, UK

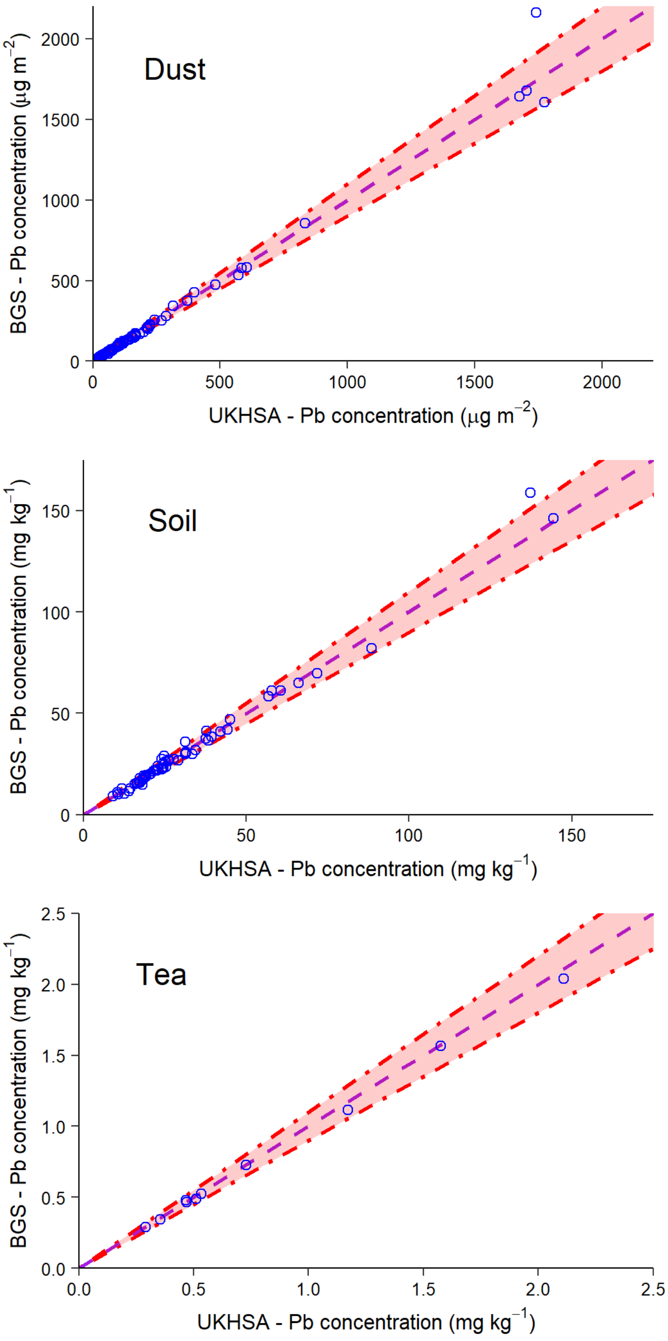

Figure S1: Comparison of Pb concentrations of household dust, soil, and tea samples as determined by BGS and UKHSA laboratories. The purple dashed line shows the line x=y while the shaded red area bound by red dot-dashed lines indicates the 10% confidence interval.

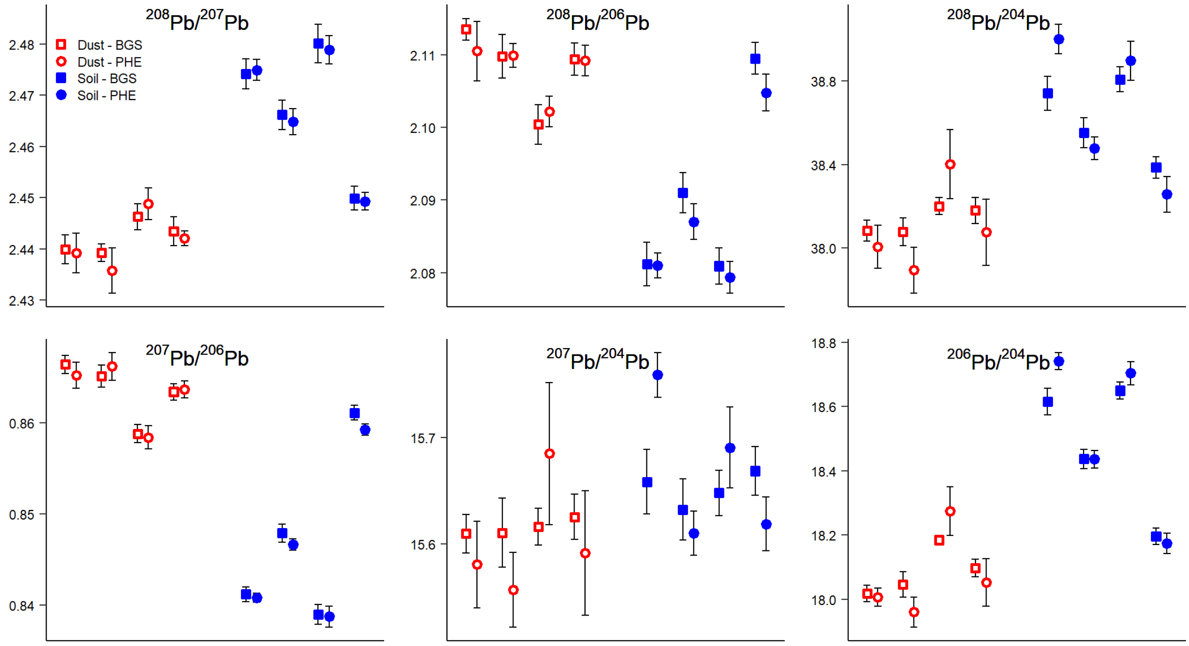

Figure S2: Pb isotope ratio data for 4 dust (open red points) and 4 soil (closed blue points) samples as determined by BGS (square points) and UKHSA (circular points) laboratories, error bars are 2 times the standard error of the mean (2SEM).

Table S1: Summary of reference materials analysed for total Pb concentration.

Certified Reference Material	Expected Concentration (mg/kg)	Mean Determined Concentration (mg/kg)		Mean Recovery (%)		Relative Standard Deviation (%)	
		BGS	UKHSA	BGS	UKHSA	BGS	UKHSA
Dust Wipes							
SRM2711a Montana II Soil (n=4)	1400	1364		97		4.6	
SRM2584 Trace Elements in Indoor Dust (n=3)	9761	9157		94		1.6	
Soils							
SRM2711a Montana II Soil (n=2)	1400	1389	1333	99	95		
BCR-2 Basalt Columbia River (n=2)	10.59	9.5	9.6	90	90		
BGS 102 Ironstone Soil (n=2)	79.4	74.2	77.1	93	97		
Teas							
GBW08505 Tea Leaves (n=4)	1.06	1.03		97		1	
Paint							
Lead in Powdered Paint SQC074 (n=7)	1960		1979		101		5
Blood							
Seronorm L-3 Trace Elements in Whole Blood (n=4)	362		363.2		100		15

Table S2: Summary of Pb isotope ratio reference material measurements performed by BGS.

Pb Isotope Ratio	206 / 207	206 / 208	206 / 204	207 / 204	207 / 206	207 / 208	208 / 204	208 / 206	208 / 204
GlenDenning UK lead ore –all matrices									
Expected	1.1787	0.4790	18.42	15.63	0.8484	0.4064	38.46	2.0876	2.4607
Mean (n=15)	1.1780	0.4787	18.4221	15.6378	0.8489	0.4064	38.4688	2.0880	2.4601
RSD (%)	0.09	0.10	0.07	0.09	0.09	0.05	0.10	0.09	0.05
Error (%)	-0.05	-0.06	0.01	0.03	0.05	-0.01	0.02	0.02	-0.03
BCR-2 – soils									
Expected	1.2007	0.4842	18.7650	15.6280	0.8328	0.4033	38.7520	2.0651	2.4796
Mean (n=4)	1.1996	0.4837	18.7825	15.6568	0.8336	0.4032	38.8263	2.0672	2.4799
RSD (%)	0.12	0.14	0.13	0.02	0.12	0.01	0.02	0.12	0.00
Error (%)	-0.09	-0.11	0.09	0.18	0.09	-0.02	0.19	0.10	0.01
